# Relationship Between Calcium and Gut Microbial Composition and Metabolic Pathways in Children with Autism

**DOI:** 10.3390/metabo16060405

**Published:** 2026-06-10

**Authors:** Jialin Li, Xinjie Xu, Huinuo Wang, Rui Gao, Bing Li, Xin You

**Affiliations:** 1 Department of Rheumatology and Clinical Immunology, Peking Union Medical College Hospital, Chinese Academy of Medical Sciences and Peking Union Medical College, Beijing 100730, China; 202393019@uibe.edu.cn (J.L.); qimingxingclinic@163.com (H.W.); 2 School of Artificial Intelligence and Data Science, University of International Business and Economics, Beijing 100029, China; 202283034@uibe.edu.cn; 3 National Infrastructures for Translational Medicine, Institute of Clinical Medicine, Peking Union Medical College Hospital, Chinese Academy of Medical Sciences and Peking Union Medical College, Beijing 100730, China; xuxin-jie@163.com; 4 National Clinical Research Center for Dermatologic and Immunologic Diseases, Ministry of Science and Technology, Beijing 100730, China; 5 State Key Laboratory of Complex Severe and Rare Diseases, Peking Union Medical College Hospital, Beijing 100730, China; 6 Key Laboratory of Rheumatology and Clinical Immunology, Ministry of Education, Beijing 100730, China; 7 Peking Union Medical Foundation, Beijing 100730, China

**Keywords:** autism spectrum disorder (ASD), hair calcium, shotgun metagenomics, gut microbiota, MetaCyc, cross-sectional study

## Abstract

**Background/Objectives:** Trace elements may influence autism spectrum disorder (ASD) severity through interactions with the gut microbiota and microbial metabolic functions, but calcium-related evidence remains limited. This cross-sectional study examined associations among hair calcium, gut microbial taxa, metabolic pathways, and behavioral phenotypes in children with ASD. **Methods:** We analyzed 183 children with ASD who had behavioral assessments, hair calcium measurements, and fecal shotgun metagenomic sequencing data. Participants in the lowest and highest calcium quartiles were first compared to characterize group-level microbiome differences. Full-sample analyses then tested associations among continuous hair calcium, microbial taxa, metabolic pathways, and behavioral measures after covariate adjustment. Benjamini–Hochberg false discovery rate correction was applied for multiple testing. **Results:** Hair calcium was positively associated with CARS, ATEC-Total, ATEC-1, and ATEC-3 scores, with the strongest associations involving ATEC-1 and ATEC-3. Alpha and beta diversity did not differ significantly between calcium quartile groups, but group-based microbiome analyses identified 63 differential species and 22 differential MetaCyc pathways. Full-sample integrated analyses connected calcium-associated microbial taxa, metabolic pathways, and ASD behavioral measures. **Conclusions:** Hair calcium was associated with ASD behavioral severity, selected gut microbial species, and microbial metabolic pathways. These findings support an association framework connecting longer-term calcium-related mineral profiles, gut microbial functional potential, and behavioral phenotypes, providing a basis for future longitudinal and multi-omics studies.

## 1. Introduction

Autism spectrum disorder (ASD) is a neurodevelopmental condition characterized by persistent difficulties in social interaction, repetitive behaviors, and restricted interests. Its global prevalence has continued to increase, but its etiological mechanisms remain incompletely understood [1]. Although genetic factors contribute substantially to ASD, environmental, metabolic, and nutritional factors have attracted increasing attention because they may influence neurodevelopment and behavioral severity [2,3,4,5]. The clinical and biological heterogeneity of ASD has prompted researchers to examine cross-system associations that may shape behavioral phenotypes.

Accumulating evidence links the gut microbiota to ASD behavioral phenotypes, potentially through the gut–brain axis [6]. This axis connects the gastrointestinal tract and central nervous system through neural, immune, and metabolic pathways. Clinical and experimental studies show that children with ASD often have gut microbial profiles that differ from those of healthy controls and relate to gastrointestinal and behavioral symptoms [7,8]. Altered gut microbiota may affect intestinal barrier function, immune responses, and microbial metabolite production, including short-chain fatty acids and other metabolites that modulate neural function [9]. Transplantation of gut microbiota from individuals with ASD into experimental animals can also induce ASD-like behavioral traits, supporting a potential role for the microbiota in neurobehavioral regulation [10,11].

Despite extensive research on the gut microbiota in ASD, no consistent microbial signature has been established. Reported alterations vary across cohorts, geographic regions, and analytical approaches, likely reflecting ASD heterogeneity, as well as differences in diet and living environment. Moreover, although gut microbial changes have been linked to behavioral phenotypes, the mechanisms by which microbial signals reach the central nervous system remain under investigation, including microbial metabolites, immune modulation, and neuroendocrine pathways.

Host nutritional status may shape gut microbial composition and behavioral phenotypes. Children with ASD commonly present with selective eating behaviors, impaired nutrient absorption, and altered trace-element profiles, which may interact with the gut microbiota to influence behavioral outcomes [12]. For example, magnesium and zinc status have been associated with ASD behavioral characteristics, whereas calcium remains relatively understudied in the context of the ASD gut–brain axis, despite its roles in neuronal excitability, synaptic transmission, and intracellular signal transduction [13,14]. Calcium is also closely linked to vitamin D metabolism, immune regulation, and metabolic homeostasis, suggesting that calcium-related status may be associated with ASD behavioral features through host physiological and microbial metabolic pathways.

Most existing studies have examined microbial composition, clinical behavioral scores, or nutritional status in isolation. Few have integrated host nutritional indicators, gut microbial functions, and ASD behavioral phenotypes within the same analytical framework [15,16]. Scalp hair provides a non-invasive matrix for assessing longer-term mineral exposure or deposition in children and is less affected by short-term physiological fluctuations than single time-point circulating measures [17]. In this study, we used hair calcium as an exploratory calcium-related mineral indicator. Because hair calcium reflects longer-term peripheral mineral exposure or deposition, whereas circulating calcium levels are maintained by tightly regulated calcium homeostatic mechanisms [18], hair calcium should not be interpreted as a direct measure of blood calcium concentration, systemic calcium nutritional status, or calcium status in neural tissues. Standardized sampling, rigorous washing, acid digestion, ICP-MS measurement, and quality control procedures were applied to support valid interpretation. We studied 183 children with ASD and integrated metagenomic sequencing with clinical behavioral assessments to analyze gut microbial diversity, differential taxa, metabolic pathways, and associations with symptom severity. This approach provides an exploratory assessment of host mineral profiles, microbial functional features, and behavioral outcomes.

## 2. Materials and Methods

### 2.1. Participants

This cross-sectional study used an existing single-center cohort of children with ASD. The final analysis included 183 participants with complete core phenotypic data, available hair calcium measurements, and fecal shotgun metagenomic sequencing data that passed quality-control requirements. Using Fisher’s z transformation for a two-sided α=0.05 test, this sample size provides approximately 80% power to detect a correlation coefficient of about 0.21, supporting adequate sensitivity for the main calcium-behavior association analyses.

The study was approved by the Institutional Review Board of Peking Union Medical College Hospital (IRB #ZS-824). Children were eligible if they had ASD confirmed by experienced psychiatrists according to the Diagnostic and Statistical Manual of Mental Disorders, Fifth Edition (DSM-V, 2013) criteria [19]; had not received antibiotics, prebiotics, or probiotics for at least four weeks before sample collection; had primary caregivers able to complete the assessment scales; and had written informed consent from a parent or legal guardian. Children with other comorbid neurological or psychiatric disorders, as confirmed by experienced clinicians or psychiatrists, were excluded. Parents or legal guardians received detailed information on the study purposes and procedures before providing consent, in accordance with the Declaration of Helsinki [20].

### 2.2. Assessment of ASD Symptoms

We assessed ASD symptom severity and behavioral characteristics using standardized rating scales:

1. Autism Behavior Checklist (ABC). The ABC consists of 57 items covering five domains: sensory responses, social relating, body and object use, language, and social and self-help skills [21]. Parents or primary caregivers completed the questionnaire based on the child’s daily behavioral performance.
2. Autism Treatment Evaluation Checklist (ATEC). The ATEC was completed by parents or caregivers and includes four subscales assessing speech/language communication, sociability, sensory/cognitive awareness, and health/physical/behavioral status [22]. Lower scores indicate fewer behavioral and functional impairments.
3. Childhood Autism Rating Scale (CARS). Trained clinical evaluators administered the CARS based on direct behavioral observation and caregiver interviews [23]. This scale is widely used in clinical and research settings to assess autism-related symptom severity and support clinical grading of ASD.

### 2.3. Hair Calcium Measurement and Calcium-Group Definition

Hair samples were collected from the scalp region using stainless steel scissors, with sampling focused on the proximal hair segment close to the scalp to reduce variation related to hair growth history and external exposure. To reduce contamination from hair-care residues and environmental particles, specimens were cut into short segments, mixed for representative subsampling, washed repeatedly with a diluted non-ionic detergent, rinsed with acetone, rinsed several times with ultrapure deionized water, rinsed again with acetone, and dried before weighing. The dried hair samples were digested using nitric acid-assisted microwave digestion and diluted with ultrapure deionized water after addition of an internal standard. Calcium concentrations were measured by inductively coupled plasma mass spectrometry (ICP-MS). Calibration verification standards, certified hair reference material, blanks, in-house controls, and spiked hair samples were included for quality control. Hair calcium levels were expressed in μg/g.

Participants were ranked according to hair calcium concentration. The low-calcium group was defined as the lowest quartile (Q1; calcium ≤227.5 μg/g; *n* = 46), and the high-calcium group was defined as the highest quartile (Q4; calcium ≥341.0 μg/g; *n* = 46). The middle two quartiles were excluded only from group-based microbiome comparisons, whereas continuous calcium values from the full sample were retained in association analyses. Clinical records indicated that none of the participants received calcium supplementation.

### 2.4. Fecal Sample Collection and DNA Extraction

Fecal samples were collected under standardized instructions provided to parents or primary caregivers. Fresh stool samples were obtained using clean containers and immediately stored at low temperature before transport to the laboratory. Upon arrival, all samples were stored at −80 °C until further processing. Microbial genomic DNA was extracted from fecal samples using the MO-BIO PowerSoil DNA extraction kit (Carlsbad, CA, USA) according to the manufacturer’s protocol. DNA was eluted in 50 μL elution buffer, and sample quality was assessed by gel electrophoresis. All extracted DNA samples were stored at −80 °C for subsequent use. Strict sterile procedures were followed throughout sample handling and DNA extraction to reduce external contamination.

### 2.5. Metagenomic Sequencing

DNA libraries were prepared using the NEBNext Ultra DNA Library Prep Kit (New England Biolabs, Ipswich, MA, USA), following the manufacturer’s workflow for DNA input amounts greater than 100 ng. Each sample was assigned a unique index, and equal amounts of indexed libraries were pooled for sequencing. Library quality was verified using the Agilent 2100 High Sensitivity DNA Kit (Agilent Technologies, Santa Clara, CA, USA), and library concentrations were quantified using the ABI 7500 Real-Time PCR System (Applied Biosystems, Waltham, MA, USA). Paired-end sequencing was performed on the Illumina HiSeq X Ten platform (Illumina, San Diego, CA, USA) with a PE150 strategy. Raw sequencing reads were processed using fastp to remove adapters and low-quality reads, and quality scores followed the Sanger/phred33/Illumina 1.8+ format. Microbial taxonomic profiles were generated using the MetaPhlAn analytical framework, and metabolic pathway abundance matrices were annotated using the HUMAnN pipeline based on the MetaCyc database. After initial abundance- and prevalence-based filtering, the full-sample dataset contained 324 microbial species and 385 MetaCyc metabolic pathways. For the group-based differential abundance analysis comparing high- and low-calcium groups, ANCOM-BC2 applied an additional prevalence threshold of 10%, retaining 254 species and 383 pathways eligible for testing.

### 2.6. Data Analysis

The analysis had two complementary components. First, participants in the lowest and highest hair-calcium quartiles were compared to evaluate whether clearly separated calcium levels corresponded to differences in overall gut microbial diversity, microbial species, and metabolic pathways. The middle two quartiles were excluded only from these group-based microbiome comparisons. Second, continuous calcium values from all 183 participants supported association analyses among hair calcium, microbial taxa, metabolic pathways, and behavioral severity measures.

Alpha diversity indices, including the Shannon index and species richness, were calculated based on the filtered microbial species abundance matrix [24,25]. Differences between the high-calcium and low-calcium groups were evaluated using the Wilcoxon rank-sum test [26]. Beta diversity was assessed using Bray-Curtis distances and visualized by principal coordinates analysis (PCoA) [27,28]. Differences in overall microbial community structure between groups were tested using permutational multivariate analysis of variance (PERMANOVA) with 999 permutations [29].

Differentially abundant microbial taxa and metabolic pathways between the high-calcium and low-calcium groups were identified using ANCOM-BC2 (Analysis of Composition of Microbiomes with Bias Correction 2) [30]. ANCOM-BC2 was selected because it accounts for the compositional nature of microbiome data, estimates bias-corrected log fold changes, and supports covariate adjustment. The ANCOM-BC2 models adjusted for age, sex, gastrointestinal symptoms, picky eating behavior, sleep disturbance, hyperactivity, allergy history, and mood variability. Features were filtered at a prevalence threshold of 10% before ANCOM-BC2 analysis.

Full-sample association analyses were performed across all 183 participants. Partial Spearman correlation analysis examined associations between hair calcium and behavioral scores after adjustment for age, sex, gastrointestinal symptoms, picky eating behavior, sleep disturbance, hyperactivity, allergy history, and mood variability. Multivariate linear regression models evaluated associations between microbial taxa and behavioral scores, between metabolic pathways and behavioral scores, and between microbial taxa and metabolic pathways. These regression models adjusted for the same covariates.

Multiple testing was controlled using Benjamini–Hochberg false discovery rate correction (BH-FDR). BH-FDR-adjusted q values were calculated within each analytical family: differential species abundance, differential pathway abundance, species-behavior associations, pathway-behavior associations, and species-pathway associations. Because this procedure controls the expected proportion of false discoveries among significant findings, it is commonly used in high-dimensional microbiome analyses involving many taxa and pathways. Unless otherwise specified, statistically significant findings were defined as BH-FDR-adjusted q ≤ 0.05.

Clinical indicators included the Childhood Autism Rating Scale (CARS), the total and subscale scores of the Autism Treatment Evaluation Checklist (ATEC), and the Autism Behavior Checklist (ABC). Statistically significant species-pathway, species-behavior, and pathway-behavior associations were integrated in a Sankey diagram to visualize links from calcium-associated gut microbial species to metabolic pathways and ASD behavioral severity.

All statistical analyses were performed using R version 4.5.1, with selected data processing and visualization tasks conducted using Python version 3.11. ANCOM-BC2 analyses were performed using the ANCOMBC package (version 2.4.0) in R. Results were visualized using boxplots, scatter plots, correlation heatmaps, and a Sankey diagram.

## 3. Results

### 3.1. Characteristics of the Enrolled Participants

This study included 183 fecal samples from children with ASD, all derived from the same research cohort. Based on the quartile distribution of hair calcium concentrations, participants in the lowest quartile (Q1; calcium ≤227.5 μg/g) were assigned to the low-calcium group (n=46), whereas those in the highest quartile (Q4; calcium ≥341.0 μg/g) were assigned to the high-calcium group (n=46) for comparative analyses. Table 1 summarizes the demographic and clinical characteristics of the two groups. Age, gastrointestinal symptoms, picky eating behavior, hyperactivity, ABC score, CARS score, and ATEC total score did not differ significantly between groups. Sex distribution differed significantly: 36/46 participants in the low-calcium group were male, compared with 20/46 in the high-calcium group (*p* = 0.001). Subsequent models included sex as a covariate.

### 3.2. Association Between Hair Calcium Levels and ASD Behavioral Phenotypes

**Figure 1:**
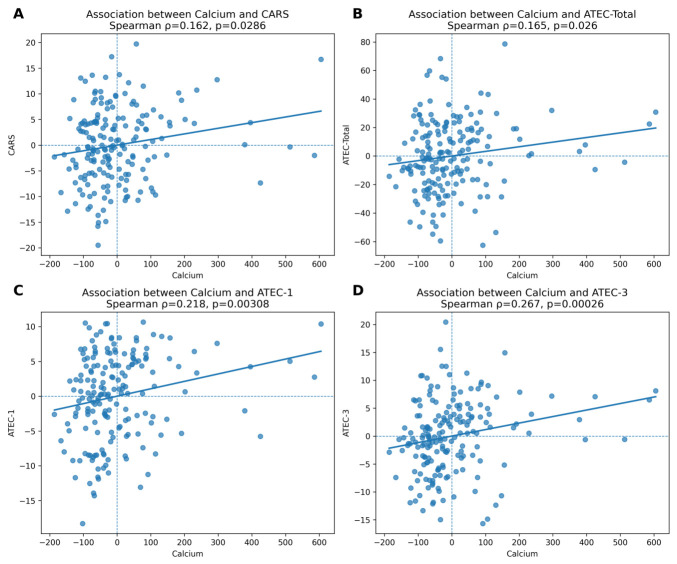
Associations between hair calcium levels and ASD behavioral measures after covariate adjustment. Scatter plots show associations between calcium concentration and behavioral scores, with fitted lines shown for visualization. Partial Spearman correlation coefficients and *p* values displayed within each panel were adjusted for age, sex, gastrointestinal symptoms, picky eating behavior, sleep disturbance, hyperactivity, allergy history, and mood variability. Hair calcium showed positive associations with (**A**) CARS (partial ρ=0.162, p=0.0286), (**B**) ATEC-Total (partial ρ=0.165, p=0.026), (**C**) ATEC-1 (partial ρ=0.218, p=0.00308), and (**D**) ATEC-3 (partial ρ=0.267, p=0.00026). Each point represents one participant.

Figure 1 shows associations between hair calcium levels and major ASD behavioral assessment scores after adjustment for age, sex, gastrointestinal symptoms, picky eating behavior, sleep disturbance, hyperactivity, allergy history, and mood variability. Partial Spearman analysis showed positive associations between hair calcium and CARS (partial ρ=0.162, p=0.0286), ATEC-Total (partial ρ=0.165, p=0.026), ATEC-1 (partial ρ=0.218, p=0.00308), and ATEC-3 (partial ρ=0.267, p=0.00026). The strongest associations involved ATEC-3 and ATEC-1.

To examine item-level behavioral features within the ATEC-3 sensory/cognitive awareness domain, we calculated partial Spearman correlations with calcium levels after adjustment for age, sex, gastrointestinal symptoms, picky eating behavior, sleep disturbance, hyperactivity, allergy history, and mood variability across the full cohort. Nine ATEC-3 items showed nominal positive correlations with calcium levels, with partial ρ ranging from 0.146 to 0.250 (all p<0.05; Table 2). After BH-FDR correction, only “draws or scribbles” remained significant at BH-FDR-adjusted q<0.05 (ρ=0.250, q=0.013); the remaining items were treated as exploratory nominal associations.

### 3.3. Overall Gut Microbial Diversity Between High- and Low-Calcium Groups

**Figure 2:**
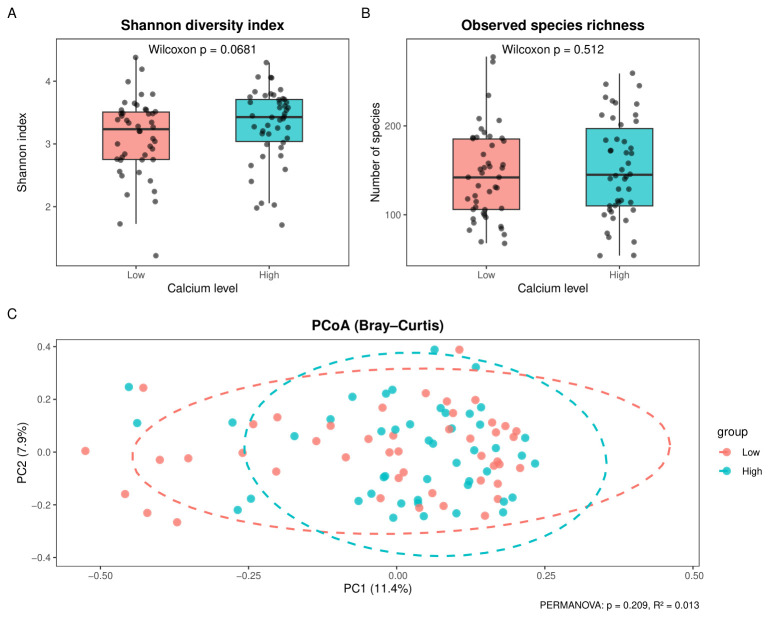
Overall gut microbial diversity between the high-calcium and low-calcium groups. (**A**) Shannon diversity index and (**B**) species richness showed largely overlapping distributions between groups, with no significant differences observed. (**C**) Principal coordinates analysis (PCoA) based on Bray-Curtis distances demonstrated substantial overlap in overall microbial community structure between the two groups. PERMANOVA further confirmed the absence of significant differences in overall microbial composition between the high-calcium and low-calcium groups.

We examined gut microbial community structure at the overall ecological level. The Shannon diversity index was slightly higher in the high-calcium group than in the low-calcium group, but the difference did not reach statistical significance (Wilcoxon rank-sum test, p=0.0681; Figure 2A). Species richness also did not differ significantly between groups (p=0.512; Figure 2B). Bray-Curtis beta diversity showed substantial overlap between the high-calcium and low-calcium groups, with no clear separation in principal coordinates analysis (PCoA) (Figure 2C). PERMANOVA showed no significant difference in overall gut microbial composition between groups (p=0.209, $R^{2}=0.013$).

### 3.4. Differential Gut Microbial Species Between High- and Low-Calcium Groups

**Figure 3:**
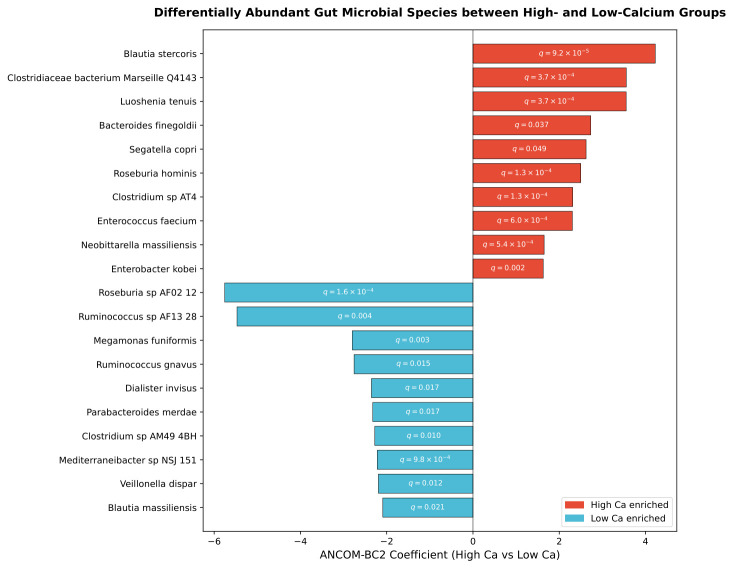
Differentially abundant gut microbial species between the high-calcium and low-calcium groups identified by ANCOM-BC2. The bar plot shows the top 20 ANCOM-BC2 coefficients for species significant at BH-FDR-adjusted q ≤ 0.05 in the covariate-adjusted model, which adjusted for age, sex, gastrointestinal symptoms, picky eating, sleep disturbance, hyperactivity, allergy history, and mood variability. Species are sorted by enrichment direction and effect size. Red bars indicate species enriched in the high-calcium group, and blue bars indicate species enriched in the low-calcium group. BH-FDR-adjusted q values are shown next to each bar.

To identify gut microbial species associated with hair-calcium group status while controlling for potential confounders, we used ANCOM-BC2 with adjustment for age, sex, gastrointestinal symptoms, picky eating behavior, sleep disturbance, hyperactivity, allergy history, and mood variability. At BH-FDR-adjusted q ≤ 0.05, ANCOM-BC2 identified 63 species with significantly different abundances between the high-calcium and low-calcium groups (Figure 3). Of these, 36 species were enriched in the high-calcium group and 27 species in the low-calcium group.

At the genus level, the high-calcium group was enriched in species related to *Clostridium*, *Eisenbergiella*, *Enterococcus*, *Blautia*, and *Faecalicatena*. In contrast, the low-calcium group showed higher abundances of species related to *Mediterraneibacter*, *Enterobacter*, *Bifidobacterium*, *Parasutterella*, and several *Clostridium*-related taxa. Representative species with the smallest BH-FDR-adjusted q values included *Collinsella aerofaciens*, *Enterocloster lavalensis*, *Escherichia coli*, *Roseburia* sp. AM59-24XD, and *Evtepia gabavorous*, which were enriched in the high-calcium group, and *Eisenbergiella massiliensis*, which was enriched in the low-calcium group.

These taxonomic differences motivated the subsequent pathway-level analysis of calcium-associated microbial metabolic functions.

### 3.5. Differential Functional Pathways Between High- and Low-Calcium Groups

**Figure 4:**
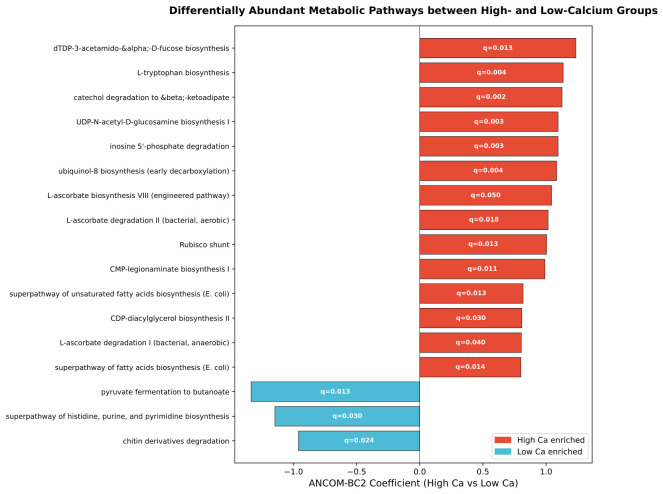
Differentially abundant metabolic pathways between the high-calcium and low-calcium groups identified by ANCOM-BC2. The bar plot shows the top 20 ANCOM-BC2 coefficients for pathways significant at BH-FDR-adjusted q ≤ 0.05 in the covariate-adjusted model, which adjusted for age, sex, gastrointestinal symptoms, picky eating, sleep disturbance, hyperactivity, allergy history, and mood variability. Pathways are sorted by enrichment direction and effect size. Red bars indicate pathways enriched in the high-calcium group, and blue bars indicate pathways enriched in the low-calcium group.

We next used ANCOM-BC2 to identify microbial metabolic pathways associated with hair-calcium group status while adjusting for age, sex, gastrointestinal symptoms, picky eating behavior, sleep disturbance, hyperactivity, allergy history, and mood variability. At BH-FDR-adjusted q ≤ 0.05, ANCOM-BC2 identified 22 MetaCyc pathways with significantly different abundances between the high-calcium and low-calcium groups (Figure 4). Of these, 19 pathways were enriched in the high-calcium group and 3 in the low-calcium group.

The high-calcium group was characterized by enrichment of pathways related to aromatic compound degradation, nucleotide degradation, amino acid metabolism, quinone biosynthesis, fatty acid biosynthesis, ascorbate metabolism, and cell wall component biosynthesis. Representative high-calcium-enriched pathways included catechol degradation to beta-ketoadipate, inosine 5’-phosphate degradation, L-tryptophan biosynthesis, ubiquinol-8 biosynthesis, fatty acid biosynthesis, and L-ascorbate degradation. The three low-calcium-enriched pathways were pyruvate fermentation to butanoate, chitin derivatives degradation, and the superpathway of histidine, purine, and pyrimidine biosynthesis.

Together, these pathway findings indicate that calcium-associated taxonomic differences were accompanied by shifts in microbial functional potential, particularly in substrate utilization, energy metabolism, amino acid and nucleotide metabolism, and microbial cell-surface biosynthesis.

### 3.6. Associations Between Differential Microbial Taxa and ASD Behavioral Severity

**Figure 5:**
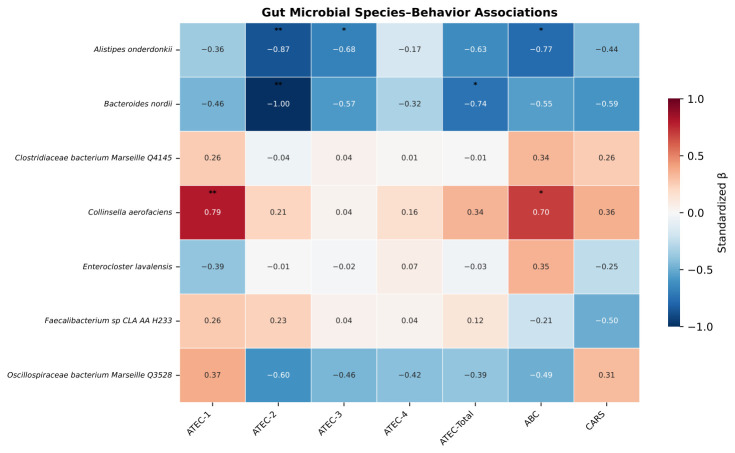
Associations between differential microbial taxa and ASD behavioral scale scores across the full sample. Multivariate linear regression analysis identified taxon-specific associations with behavioral dimensions after adjustment for age, sex, gastrointestinal symptoms, picky eating, sleep disturbance, hyperactivity, allergy history, and mood variability. Colors indicate standardized regression coefficients. Asterisks indicate associations that remained statistically significant after BH-FDR correction across all tested taxon-behavior pairs: * BH-FDR-adjusted q < 0.05 and ** BH-FDR-adjusted q < 0.01.

We evaluated associations between differential microbial taxa and ASD behavioral scale scores across the full sample using multivariate linear regression models adjusted for age, sex, gastrointestinal symptoms, picky eating behavior, sleep disturbance, hyperactivity, allergy history, and mood variability (Figure 5). The heatmap shows standardized regression coefficients for seven microbial species across ATEC subdomains, ATEC-Total, ABC, and CARS. After BH-FDR correction, *Collinsella aerofaciens* was positively associated with ATEC-1 (Speech/Language/Communication; standardized β=0.24) and ABC scores (standardized β=0.87). In contrast, *Alistipes onderdonkii* was negatively associated with ATEC-2 (Sociability; standardized β=−0.28), ATEC-3 (Sensory/Cognitive Awareness; standardized β=−0.18), and ABC scores (standardized β=−0.87). *Bacteroides nordii* was negatively associated with ATEC-2 (standardized β=−0.47) and ATEC-Total (standardized β=−1.14). These findings indicate taxon-specific and behavioral-domain-specific associations between calcium-related gut microbial species and ASD behavioral phenotypes.

### 3.7. Associations Between Differential Metabolic Pathways and ASD Behavioral Severity

**Figure 6:**
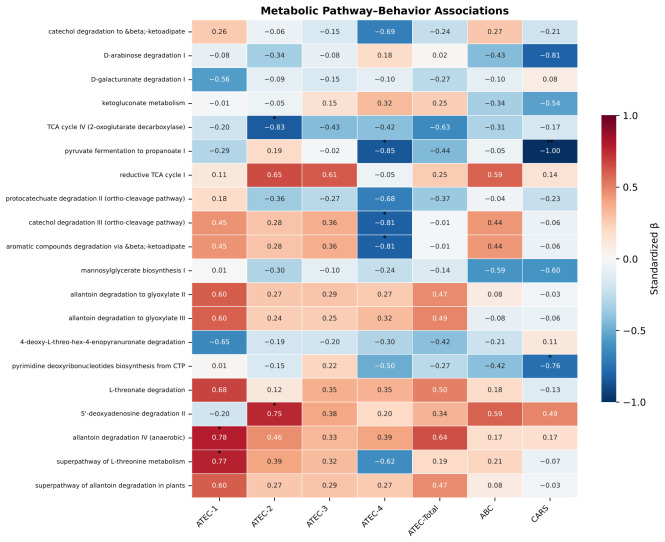
Associations between differential metabolic pathways and ASD behavioral symptom severity across the full sample. Multivariate linear regression analysis identified pathway-specific associations with behavioral dimensions after adjustment for age, sex, gastrointestinal symptoms, picky eating, sleep disturbance, hyperactivity, allergy history, and mood variability. Colors indicate standardized regression coefficients across ATEC subdomains, ATEC-Total, ABC, and CARS. Asterisks indicate associations that remained statistically significant after BH-FDR correction across all tested pathway-behavior pairs: * BH-FDR-adjusted q < 0.05 and ** BH-FDR-adjusted q < 0.01.

Across the full sample, we used multivariate linear regression models to assess associations between differential metabolic pathways and ASD behavioral symptom severity, adjusting for age, sex, gastrointestinal symptoms, picky eating behavior, sleep disturbance, hyperactivity, allergy history, and mood variability (Figure 6). The heatmap shows standardized regression coefficients for metabolic pathways across ATEC subdomains, ATEC-Total, ABC, and CARS. BH-FDR-significant associations were concentrated in selected behavioral domains. TCA cycle IV (2-oxoglutarate decarboxylase) and 5’-deoxyadenosine degradation II were associated with ATEC-2, with opposite directions of effect. Pyruvate fermentation to propanoate I, catechol degradation III, and aromatic compounds degradation via beta-ketoadipate were negatively associated with ATEC-4, whereas allantoin degradation IV (anaerobic) and the superpathway of L-threonine metabolism were positively associated with ATEC-1. Pyruvate fermentation to propanoate I and pyrimidine deoxyribonucleotides biosynthesis from CTP were negatively associated with CARS. These results indicate pathway-specific and behavioral-domain-specific associations, with several degradation, fermentation, TCA-related, nucleotide-related, and amino acid-related pathways linked to ASD behavioral measures.

### 3.8. Associations Between Microbial Taxa and Differential Metabolic Pathways

**Figure 7:**
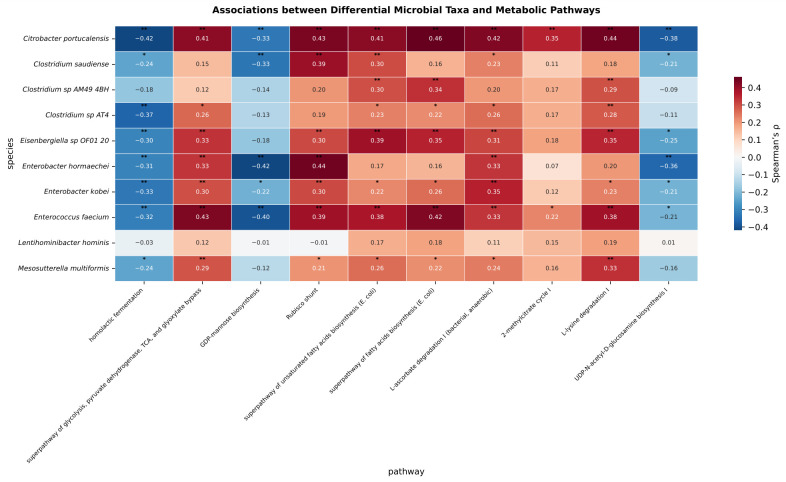
Associations between differential microbial taxa and metabolic pathways after covariate adjustment. The heatmap shows standardized regression coefficients from multivariate linear regression models between the top 10 ANCOM-BC2 significant species and top 10 significant metabolic pathways after adjustment for age, sex, gastrointestinal symptoms, picky eating, sleep disturbance, hyperactivity, allergy history, and mood variability. Colors indicate standardized regression coefficients. Asterisks indicate associations that remained statistically significant after BH-FDR correction across all tested species-pathway pairs: * BH-FDR-adjusted q < 0.05 and ** BH-FDR-adjusted q < 0.01.

We examined associations between differential microbial taxa and differential metabolic pathways using multivariate linear regression models adjusted for age, sex, gastrointestinal symptoms, picky eating behavior, sleep disturbance, hyperactivity, allergy history, and mood variability (Figure 7). The heatmap shows standardized regression coefficients between the top differential microbial species and metabolic pathways. Several taxa, including *Citrobacter portucalensis*, *Enterococcus faecium*, *Enterobacter hormaechei*, *Enterobacter kobei*, *Eisenbergiella* sp. OF01 20, *Clostridium saudiense*, and *Clostridium* sp. AT4, showed coordinated associations with pathways involved in fermentation, central carbon metabolism, fatty acid biosynthesis, amino acid degradation, and microbial cell-wall-related biosynthesis.

### 3.9. Integrated Associations Among Hair Calcium Levels, Gut Microbiota, Metabolic Pathways, and ASD Behavioral Phenotypes

**Figure 8:**
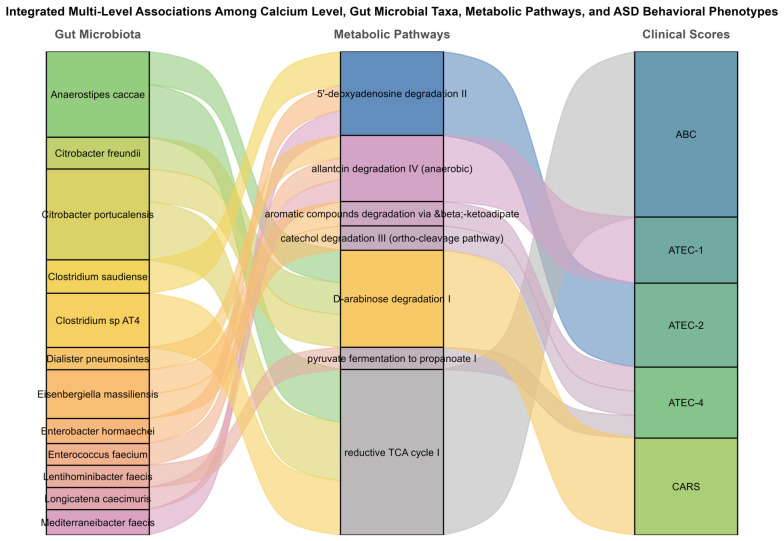
Integrated associations among calcium-associated gut microbial taxa, metabolic pathways, and ASD behavioral phenotypes. The Sankey diagram summarizes statistically significant links identified across full-sample covariate-adjusted analyses. The left column shows differential microbial taxa, the middle column shows differential metabolic pathways, and the right column shows ASD behavioral scale scores. Links indicate statistically significant associations connecting microbial taxa with metabolic pathways and pathways with behavioral scores.

Calcium-associated microbial taxa, metabolic pathways, and ASD behavioral scale scores were integrated in a Sankey diagram (Figure 8). The diagram summarizes statistically significant links from the full-sample covariate-adjusted analyses and illustrates a multi-level association pattern connecting microbial taxa, metabolic pathways, and behavioral severity measures. For example, 5’-deoxyadenosine degradation II and pyruvate fermentation to propanoate I appeared as intermediate nodes linking calcium-associated taxa with behavioral domains including ATEC-2, ATEC-4, and CARS.

## 4. Discussion

In this cohort of children with ASD, hair calcium was associated with behavioral severity, selected gut microbial taxa, and microbial metabolic pathways. Children in the high- and low-calcium quartiles did not differ in overall microbial diversity or community structure, but they differed in specific species and MetaCyc pathways after adjustment for demographic and clinical covariates. The strongest calcium-behavior associations involved ATEC-1 and ATEC-3, suggesting that calcium-related biological or environmental variation may align more closely with communication and sensory or cognitive domains than with global behavioral measures alone. This interpretation is consistent with evidence that mineral status, nutritional factors, gut microbial features, and neurodevelopmental phenotypes may interact in ASD [5,12,15,16]. The integrated Sankey analysis further placed calcium-associated taxa and pathways within a broader network of associations with ASD behavioral scores.

These findings fit the heterogeneous ASD gut microbiome literature. Previous studies have reported inconsistent alpha- and beta-diversity findings, whereas taxon-level and functional differences are more often observed across cohorts [6,7,8,31]. The absence of a strong diversity signal in the present study therefore does not exclude biologically relevant microbial variation. Instead, hair-calcium-defined groups differed at a more specific metagenomic level. The enriched taxa include organisms with different substrate utilization, fermentation, organic acid production, carbohydrate metabolism, and amino acid-related capacities, providing a plausible ecological basis for the functional pathway differences observed between groups [32,33,34].

The pathway findings suggest that calcium-related microbial differences were accompanied by shifts in functional potential. Most differential pathways were enriched in the high-calcium group and involved aromatic compound degradation, nucleotide degradation, amino acid metabolism, quinone biosynthesis, fatty acid biosynthesis, ascorbate metabolism, and cell wall component biosynthesis. These functions may reflect differences in microbial substrate processing, redox metabolism, membrane or cell surface biosynthesis, and energy-related activity [33,35,36]. Low-calcium-enriched pathways, including pyruvate fermentation to butanoate, chitin derivatives degradation, and the superpathway of histidine, purine, and pyrimidine biosynthesis, indicate a different functional profile involving fermentation, polysaccharide derivative degradation, and nucleotide or amino acid biosynthesis. Fermentation-derived metabolites, including propionate and butyrate, have been implicated in host metabolism and nervous system function [37,38,39]. Amino acid, biogenic amine, and nucleotide-related pathways are also relevant to ASD because they may affect neurotransmitter precursor availability, immune regulation, and cellular energy balance [40,41,42]. Because shotgun metagenomics measures functional potential rather than metabolite flux, these pathway-level findings should be interpreted as candidate functional signatures for future metabolomic validation.

The association analyses connected these microbial features with ASD behavioral domains. *Collinsella aerofaciens* was positively associated with ATEC-1 and ABC scores, whereas *Alistipes onderdonkii* and *Bacteroides nordii* showed negative associations with selected ATEC subdomains or total scores. Several pathways, including TCA cycle IV, 5’-deoxyadenosine degradation II, pyruvate fermentation to propanoate I, catechol degradation III, aromatic compound degradation via beta-ketoadipate, allantoin degradation IV, the superpathway of L-threonine metabolism, and pyrimidine deoxyribonucleotide biosynthesis from CTP, were also associated with specific behavioral measures. Rather than indicating a uniform relationship between the microbiome and ASD severity, these domain-specific associations suggest that different taxa and pathways may relate to different behavioral dimensions. This pattern is consistent with ASD clinical heterogeneity and with models in which gut microbial functions may influence neurodevelopmental phenotypes through metabolic, immune, and neuroendocrine routes [6,9,10,11,36,43].

The integrated network helps organize these layered associations into a framework linking microbial taxa, metabolic pathways, and behavioral phenotypes. It does not demonstrate mediation or causality, but it aligns with emerging multi-omics approaches that integrate nutrition, microbial community structure, functional capacity, metabolite profiles, and neurodevelopmental outcomes [15,16,34,35].

Scalp hair provides a non-invasive matrix that can capture longer-term mineral exposure or deposition in children, whereas circulating calcium can fluctuate with short-term physiological regulation [44,45]. Hair-based element analysis has been used in pediatric ASD studies of calcium and other trace elements, and its interpretation depends on standardized sampling, washing, digestion, instrumental analysis, and quality control [46,47]. Potential influences from external contamination, hair-care products, environmental exposure, sampling site, and laboratory preprocessing were addressed through proximal scalp-hair sampling, repeated detergent and solvent washing, ultrapure deionized water rinsing, acid digestion, ICP-MS measurement, and reference and control materials [48]. Under these standardized procedures, hair calcium was a suitable exploratory matrix for stratifying children by longer-term calcium-related mineral profiles.

Nutritional and gastrointestinal factors may influence the observed associations because they can affect gut microbial composition, calcium-related mineral profiles, and behavioral symptoms. Several potential sources of confounding were therefore considered [12,49,50,51,52]. First, calcium supplementation could directly alter calcium-related measures and indirectly affect microbial metabolism; however, clinical records indicated that none of the participants received calcium supplementation during the study period. Second, habitual diet can shape both gut microbiota and mineral intake [53,54]. Although detailed dietary records were not available, the participants were recruited from the same clinical cohort, and children with recent antibiotic, prebiotic, or probiotic exposure were excluded for at least four weeks before sample collection to reduce major microbiome-modifying influences. Third, gastrointestinal symptoms may affect nutrient absorption, microbial ecology, and behavioral presentation [55,56,57]. The low- and high-calcium quartile groups did not differ significantly in gastrointestinal symptoms, and gastrointestinal symptoms were included as a covariate in the main models. Fourth, picky or selective eating is common in ASD and may influence dietary calcium intake and microbial substrate availability. Picky eating behavior was comparable between groups and was also adjusted for in the microbiome and behavioral association models. Together with adjustment for age, sex, sleep disturbance, hyperactivity, allergy history, and mood variability, these procedures reduced measured differences that could confound associations among hair calcium, gut microbial features, and ASD behavioral scores. Nevertheless, residual confounding remains possible because quantitative dietary intake, broader non-calcium supplement exposure, and biochemical markers of mineral metabolism were not comprehensively collected.

Several limitations should guide interpretation. The cross-sectional, single-cohort design does not establish temporal ordering or causality among hair calcium, microbial features, metabolic pathways, and behavioral phenotypes, and the findings require validation in independent cohorts. The relatively restricted pediatric age distribution reduced developmental heterogeneity within this cohort, but future studies should test whether these associations extend to broader age ranges and typically developing comparison groups.

Measurement and confounding constraints also remain. The significant sex imbalance between the low- and high-calcium groups should be considered when interpreting the microbiome and behavioral association results, because sex-related differences may influence gut microbial composition and ASD behavioral presentation. Although the analyses adjusted for sex, gastrointestinal symptoms, picky eating behavior, sleep disturbance, hyperactivity, allergy history, and mood variability, residual confounding may remain because detailed dietary composition, environmental exposure, supplement use, and mineral metabolism measures were not comprehensively characterized.

## 5. Conclusions

In this cross-sectional cohort of children with ASD, hair calcium was associated with behavioral severity, selected gut microbial species, and microbial metabolic pathways. Group-based microbiome comparisons identified calcium-associated microbial and functional features, and full-sample analyses linked selected taxa and pathways with ASD behavioral measures. The integrated network supports an association framework connecting hair calcium, gut microbial functional potential, and behavioral phenotypes, without establishing causality. Longitudinal studies integrating standardized hair mineral assessment, dietary and supplement records, environmental exposure information, and metabolomics are needed to validate these findings.

## Figures and Tables

**Table 1:** Baseline demographic and clinical characteristics of children with ASD in the low-calcium and high-calcium groups.

Variable	Low Ca Group (n=46)	High Ca Group (n=46)	*p* Value
Age (years)	4.02 ± 1.13	4.57 ± 1.65	0.066
Sex (male, %)	78.3% (36/46)	43.5% (20/46)	0.001 **
Gastrointestinal symptoms (%)	86.7% (39/45)	89.1% (41/46)	0.758
Picky eating (%)	90.9% (40/44)	84.8% (39/46)	0.523
Hyperactivity (%)	95.7% (44/46)	93.5% (43/46)	1.000
ABC score	64.72 ± 24.72	70.33 ± 27.45	0.306
CARS score	37.98 ± 7.27	40.65 ± 7.19	0.080
ATEC total score	80.00 ± 23.00	85.89 ± 26.32	0.256
Hair calcium	194.85 ± 26.47	453.87 ± 148.55	<0.001 ***

Data are presented as mean ± SD or percentage (*n*/N), as appropriate. ASD, autism spectrum disorder; ABC, Autism Behavior Checklist; CARS, Childhood Autism Rating Scale; ATEC, Autism Treatment Evaluation Checklist; Q1, first quartile; Q4, fourth quartile. ** *p* < 0.01, *** *p* < 0.001.

**Table 2:** Associations between hair calcium levels and individual items of the ATEC-3 sensory/cognitive awareness subscale.

ATEC-3 Item	Partial ρ	*p* Value	BH-FDR *q*
Draws or scribbles **	0.250	0.001	0.013
Imagination *	0.179	0.016	0.073
Initiative *	0.175	0.018	0.073
Looks at pictures/TV *	0.174	0.018	0.073
Awareness of danger *	0.172	0.020	0.073
Responds to own name *	0.166	0.025	0.073
Uses toys appropriately *	0.163	0.027	0.073
Dresses independently *	0.157	0.033	0.079
Notices changes in surroundings *	0.146	0.049	0.103

Partial Spearman correlations adjusted for age, sex, gastrointestinal symptoms, picky eating behavior, sleep disturbance, hyperactivity, allergy history, and mood variability. BH-FDR-adjusted *q* values were computed using the Benjamini–Hochberg method across all 18 ATEC-3 items. Only items with nominal *p* < 0.05 are shown; after BH-FDR correction, only “Draws or scribbles” remained significant at *q* < 0.05. The remaining items are reported as exploratory nominal associations. * *p* < 0.05, ** *p* < 0.01.

## Data Availability

The raw sequence data reported in this paper have been deposited in the Genome Sequence Archive [58] in National Genomics Data Center [59], China National Center for Bioinformation/Beijing Institute of Genomics, Chinese Academy of Sciences (GSA: CRA044389) that are publicly accessible at https://ngdc.cncb.ac.cn/gsa.

## Abbreviations

The following abbreviations are used in this manuscript:

ABC	Autism Behavior Checklist
ANCOM-BC2	Analysis of Composition of Microbiomes with Bias Correction 2
ASD	Autism Spectrum Disorder
ATEC	Autism Treatment Evaluation Checklist
CARS	Childhood Autism Rating Scale
FDR	False Discovery Rate
ICP-MS	Inductively Coupled Plasma Mass Spectrometry
MetaCyc	Metabolic pathway database
PCoA	Principal Coordinate Analysis
PERMANOVA	Permutational Multivariate Analysis of Variance
Q1	First quartile
Q4	Fourth quartile
